# Supplementary material for: Cryptococcal Hsf3 controls intramitochondrial ROS homeostasis by regulating the respiratory process
Source: Nat Commun. 2022 Sep 15;13:5407. doi: 10.1038/s41467-022-33168-1 (PMC9477856; doi:10.1038/s41467-022-33168-1)
Supplement: Supplementary file 1 — Supplementary Information [file 41467_2022_33168_MOESM1_ESM.pdf]

Supplementary Information

Cryptococcal Hsf3 controls intramitochondrial ROS homeostasis via regulating the respiratory process

Xindi Gao, Yi Fu, Shengyi Sun, Tingyi Gu, Yanjian Li, Tianshu Sun, Hailong Li, Wei Du, Chenhao Suo, Chao Li, Yiru Gao, Yang Meng, Yue Ni, Sheng Yang, Tian Lan, Sixiang Sai, Jiayi Li, Kun Yu, Ping Wang, Chen Ding

This PDF file includes:

1. Supplementary Methods
2. Supplementary Figures 1 to 11
3. Supplementary References

1. Supplementary Methods

The manipulations of strains

Yeast manipulation and protocols were applied as described elsewhere [1]. The *CnHSF3* gene (*CNAG_04036*) was disrupted by the homologous replacement of its open reading frame with a piece of DNA containing a dominant drug resistance gene marker. In the first round of PCR, the ssy29/ssy30 and ssy31/ssy32 primer pairs were used to amplify the 5' and 3' flanking regions of the *CnHSF3* gene, respectively. The NEO dominant marker was amplified from the *pHA-NEO* vector using the M13F/M13R primer pair. In the second round of PCR, a continuous fragment was created using three gel-extracted DNA fragments from the first-round PCR as templates, and the *Cnhsf3Δ::NEO* construct was amplified using the ssy29/ssy32 primer pair. The *C. neoformans strain* H99 was biolistically transformed using the deletion cassette. To identify the desired *Cnhsf3Δ*, diagnostic PCR was performed using the ssy18/ssy19 primer pair.

To construct the C. neoformans *CnHSF3:: Cnhsf3Δ* strain, the *Cn*HSF3 upstream and coding sequences were PCR-amplified from genomic DNA using the x-up-F3 and x-orf-R3 primers. After digestion with *Sal*I and *Sma*I, the digest products were cloned into *pFLAG-NAT*, constructed by inserting

*BamH*I-*Spe*I to the *CnHSF3* downstream sequence using the x-dw-F3 and x-dw-R3 primers. The cassette amplified from the final *pFLAG-NAT* using the x-up-F3 and x-dw-R3 primers was biolistically transformed into the *Cnhsf3Δ::NEO* strain. Diagnostic PCR using the GXD158/GXD159 primer pair was performed, and immunoblot analysis of *Cn*Hsf3-FLAG was performed using the anti-Flag mouse monoclonal antibody.

To construct *CnHSF3-FLAG* strains, a *TEF* promoter and *CnHSF3* open reading frame were amplified from C. neoformans genomic DNA using the Xin-T-F/Xin-T-R and Xin-O-F/Xin-O-R primer pairs. The *TEF* promoter and *CnHSF3* open reading frame were overlapped and cloned into *pFLAG-NAT*, constructed by inserting *CnHSF3* upstream and downstream. The cassette amplified from the final *pFLAG-NAT* harboring intact *CnHSF3* open reading frames using x-up-F3 and x-dw-R3 primers was biolistically transformed into the *Cnhsf3Δ::NEO* strain. Diagnostic PCR was performed using the GXD158/GXD159 primer pair, and immunoblot analysis of *Cn*Hsf3 was performed using the anti-Flag mouse monoclonal antibody.

To construct *CnHSF1-FLAG* strains, a TEF *promoter* was amplified from C. neoformans genomic DNA using the GXD285 and GXD286 primers. The PCR product was cloned into *pFLAG-NAT* between the *BamH*I and *Spe*I sites. The

NAT-TEFp cassette was then amplified from the constructed plasmid using the GXD287 and GXD289 primers. During the second round of PCR, GXD290/GXD291 and GXD293/GXD294 primer pairs were used to amplify the 1-kb promoter region and coding sequence of the *CnHSF1* gene with the FLAG tag. In the third round of PCR, an overlapped fragment was created, the *CnHSF1-FLAG* construct was amplified using the GXD290/GXD294 primer pair, and three gel-extracted DNA fragments from the first and second rounds were used as templates for overlapping PCRs. C. neoformans strain H99 was biolistically transformed using the *CnHSF1-FLAG* cassette. To identify the desired strain, diagnostic PCR was performed using the GXD290/GXD294 primer pair, and qRT-PCR was performed using the GXD162/GXD163 primer pair. Immunoblot analysis of *Cn*Hsf1-FLAG was performed using the anti-Flag mouse monoclonal antibody.

To generate the *CnHSF3-GFP* strain, the open reading frame of *CnHSF3*, amplified using the ssy152/ssy153 primer pair and GFP-amplified using the ssy154/ssy115 primer pair, were overlapped using the ssy152/ssy115 primer pair. The cassette was cloned at the *Pac*I site of *pHYG* plasmid which was then biolistically transformed into the H99 strain.

The *GalP-CnHSF1* strain was constructed similarly as the *CnHSF1-FLAG* strain. The *GAL* promoter was amplified from *C. neoformans* genomic DNA using the GXD628 and GXD629 primers and was cloned into *pFLAG-NAT* between the *BamH*I and *Spe*I sites. The *NAT-GalP* cassette was then amplified using the GXD287 and GXD823 primers. For the second round of PCR, the 1-kb promoter region and coding sequence of the *CnHSF1* gene with a FLAG tag sequence was amplified using GXD290/GXD291 and GXD408/GXD294 primer pairs. Finally, three gel-extracted DNA fragments from the first and second-round PCRs were used as templates for overlapping PCR to generate the *GalP-CnHSF1* integration cassette using the GXD290/GXD294 primer pair. The resulting PCR products were then purified and biolistically transformed into *C. neoformans* strain H99. To identify the desired strain, immunoblot analysis using the *GalP-CnHSF1* strain grown in YPD or YPGal liquid media was performed using the anti-Flag mouse monoclonal antibody.

To generate the *hHSF5-FLAG* strain, the cDNA sequence of hHsf5 was amplified using GXD850/ GXD853 primer pair. The reconstructed plasmid was generated as described for *TIM44-FLAG* strain construction. The 5’ flanking region and the *TEF1* promoter were amplified using the 4466-my/ GXD681 pair, and then the cDNA sequence of hHsf5 was overlapped with the

5’ flanking region and the *TEF1* promoter using the 4466-my/GXD853 primer pair. The PCR products were cloned into the *pHA-NEO* with the 3’ flanking region between the *Apa*I and *Sma*I sites. Finally, the cassette was amplified using the 4466-my/4471-my primer pair followed by biolistic transformation into the *Cnhsf3*Δ::NEO strain. Immunoblot analysis of hHSF5-FLAG was performed using the anti-Flag mouse monoclonal antibody.

To detect the localization of *Cn*Hsf3 in mitochondria, the *TIM44-FLAG* strain was constructed. An intact *TIM44* gene was amplified using the GXD713/GXD714 primer pair. The “safe haven” site was used to integrate the target gene (Arras et al., 2015), then its 3’ flanking region was amplified using the 4470-my/4471-my primer pair, and this was then cloned into *pHA-NEO* between the *Sac*II and *Sac*I sites. The 5’ flanking region was amplified using the 4466-my/4467-my primer pair, the *TEF1* promoter was amplified using the 4468/GXD681 primer pair, and the open reading frame of *TIM44* was overlapped using the 4466-my/GXD714 primer pair, then was cloned into *pHA-NEO* with the 3’ flanking region between the *Apa*I and *Sma*I sites. Finally, the cassette was amplified using the 4466-my/4471-my primer pair followed by biolistic transformation into the *TEF1-CnHSF3* strain. To identify the desired strain, diagnostic PCR was performed using the 4466-

my/GXD714 primer pair, and immunoblot analysis of Tim44 protein expression was performed using anti-HA (C29F4) rabbit mAb.

The *CNAG_09000* overexpressing strain was constructed in the same way. Briefly, the open reading frame products of *CNAG_09000* were amplified using the GXD756/GXD757 primer pair and was overlapped with the 5’ flanking region of the safe haven site and TEF1 promoter, which was then cloned into *pHA-NEO* with the 3’ flanking region between the *Apa*I and *Sma*I sites. The PCR products, obtained using the 4466-my/4471-my primer pair, were biolistically transformed into the *QCR9* overexpressing strain. Diagnostic PCR was performed using the 4466-my/4471-my primer pair, and qRT-PCR was performed using the GXD588/GXD589 primer pair.

To construct the *SOD1* and *SOD2* overexpressing strains, the *SOD1* and *SOD2* coding sequences were amplified using the GXD542/GXD542 and GXD543/GXD544 primer pairs and were cloned at the *Pac*I site of the *pHYG* plasmid (containing a hygromycin resistance marker under the control of the C. neoformans ACT1 promoter). The cloned plasmids were biolistically transformed into the *Cnhsf3*Δ. The constructed *SOD1* and *SOD2* overexpressing strains were identified using qRT-PCR and the

GXD614/GXD615 and GXD616/GXD617 primer pairs. Immunoblot analysis was performed using the anti-Flag mouse monoclonal antibody.

To construct the *QCR9* overexpressing strains, the *QCR9* coding sequence was amplified using the GXD682 and GXD683 primers, and the TEF1 promoter was amplified using the ssy57 and GXD681 primers. The two PCR products were overlapped using the ssy57 and GXD683 primers, and the resulting PCR product was digested using *Hind*III and *Sma*I. The digest products were cloned into *pFLAG-NAT*, and the cassette was amplified using the x-up-F3 and x-dw-R3 primers. The PCR product was biolistically transformed into the *Cnhsf3Δ::NEO* strain. Diagnostic PCR was performed using the x-up-F3 and x-dw-R3 primer pair, and qRT-PCR was performed using the GXD532/GXD533 primer pair.

To generate the *NDUFA5* overexpressing strain, the upstream sequence and coding sequence of *NDUFA5* were amplified from *C. neoformans* genomic DNA using the GXD725/GXD726 and GXD727/GXD728 primer pairs. The upstream sequence and coding sequence of *NDUFS6* were amplified using the GXD721/GXD722 and GXD723/GXD724 primer pairs. The TEF1 promoter was amplified using the GXD285 and GXD286 primers and then was cloned into *pHA-NEO* between the *BamH*I and *Spe*I sites. To construct

the *NDUFA5* overexpressing strain, the three gel-extracted DNA fragments (the upstream sequence of *NDUFA5*, the *NEO-TEFp* cassette, and the coding sequence of *NDUFA5*) were pooled and overlapped using the GXD725/GXD728 primer pair. The *NDUFS6* overexpressing strain was similarly generated: the three gel-extracted DNA fragments were the upstream sequence of *NDUFS6*, the *NEO-TEFp* cassette, and the coding sequence of *NDUFS6*, and the primer pair was GXD721/GXD724. The final cassettes were biolistically transformed into *QCR9* overexpressing strains and the *Cnhsf3Δ::NEO* strain, respectively. All strains were identified by performing diagnostic PCR using the GXD725/GXD728 and GXD721/GXD724 primer pairs and qRT-PCR using the GXD731/GXD732 and GXD729/GXD730 primer pairs, respectively.

To generate a strain without the *Cn*Hsf3 mitochondrion targeting signal (MTS), the reconstructed plasmid previously used to generate the *CnHSF3:: Cnhsf3Δ* strain was used as a template and was amplified using the GXD802 and GXD803 primer pair The linear fragment was then restored to an integral plasmid using a Blunting Kination Ligation Kit (Takara) according to manufacturer instructions. The cassette was amplified from the reconstructed plasmid using the x-up-F3 and x-dw-R3 primer pair then was biolistically transformed into the *Cnhsf3Δ::NEO* strain. This transformed strain was then

transformed using the *TIM44-HA* cassette. To identify the strain, diagnostic PCR was performed using the GXD158/GXD159 primer pair, then immunoblot analysis was performed. The *CnHSF3 (NLSmut)*, *CnHSF3 (MTSmut)*, and *CnHSF3C130A* strains were constructed in the same way using the GXD818/GXD819, GXD816/GXD817, and GXD769/GXD770 primer pairs, respectively. PCR identification was performed using the GXD158/GXD159 primer pair, and immunoblot analysis was performed using the anti-Flag mouse monoclonal antibody.

To characterize the MTS and NLS mutation of *Cn*Hsf3, the cassette was amplified using the GXD816/GXD817 and GXD818/GXD819 primer pair from the plasmid used to construct the *CnHSF3-FLAG* strains. The resulting PCR product was biolistically transformed into the *Cnhsf3*Δ::*NEO* strain, and the resulting transformants were transformed using the *TIM44-HA* cassette. To identify the strain, immunoblot analysis was performed using the anti-Flag mouse monoclonal antibody.

To characterize the NLS mutation of *Cn*Hsf3, the reconstructed plasmid used to construct the *CnHSF3-GFP* strain was used as a template and was amplified using the GXD818 and GXD819 primers. An integral plasmid was generated by cyclizing the linear PCR product and then transforming it into

the *Cnhsf3*Δ strain. Similarly, The *CnHSF3(NLSmut)-GFP* strain and *CnHSF3 C130A-GFP* were constructed using GXD816/GXD817 and GXD769/GXD770 primer pairs, respectively. The open reading frame of mCherry was amplified using GXD851 and GXD852 primers and inserted into the previously reconstructed plasmid carrying 5’ and 3’ flanking region of the safe haven site and the open reading frame of TIM44. The cassette was obtained using the 4466-my/4471-my primer pair and biolistically transformed into the *CnHSF3-GFP* and *CnHSF3(NLSmut)-GFP* strain, respectively.

To validate the effects of Sod1 and Sod2 on the DNA binding of *Cn*Hsf3, either *SOD1* or *SOD2* was overexpressed in the *CnHSF3-FLAG* strain as described for *CNAG_09000.* The open reading frame of *SOD1* or *SOD2* was amplified using the GXD689/GXD690 or GXD691/GXD692 primer pairs, respectively, and were overlapped with the 5’ flanking region of the safe haven site and TEF1 promoter, then cloned into *pHA-NEO* using the 3’ flanking region between the *Apa*I and *Sma*I sites. The PCR products, obtained using the 4466-my/4471-my primer pair, were biolistically transformed into the *CnHSF3-FLAG* strain. Diagnostic PCR was performed using the 4466-my/4471-my primer pair, and qRT-PCR was performed using the GXD614/GXD615 and GXD616/GXD617 primer pairs, respectively.

Electrophoretic mobility shift assay (EMSA)

EMSA was performed according to the Chemiluminescent EMSA Kit (Beyotime Biotechnology, GS009) protocol. The 5’ biotinylated oligo (forward strand of oligo 3; GXD798) and non-biotinylated oligo (a reverse strand of oligo 3; GXD799) were heated to 95°C followed by cooling to room temperature to allow the formation of the double-stranded DNA probe. In addition, the PCR products amplified by 5’ biotinylated primer GXD753 and non-biotinylated primer GXD587 were used as the probe. The *HSP70* and *HSP90* promoter fragments amplified using primer pairs GXD703/GXD704 and GXD482/GXD483, respectively were used as competitive probes for *Cn*Hsf3-part1. For nonspecific competitor or cold probe, a 100-fold non-labeled DNA probe was added. Briefly, purified *Cn*Hsf3-part1 or NaClO-treated *Cn*Hsf3-part1 protein was incubated with probes for 20 min at RT. The mixture was separated using 4% PAGE electrophoresis, transferred onto a nitrocellulose membrane, and crosslinked using a portable UV detector at 254 nm for 10 min. The nitrocellulose membrane was then blocked for 15 min with Blocking Solution and incubated with Streptavidin-HRP Conjugate for 15 min. The membrane was washed three times with Washing Buffer gently for 5 min, followed by incubating with a Balance Solution for 5 min. Immunoblotting assays were performed and the signal was captured using a ChemiDoc XRS+ (Bio-Rad).

RNA-seq and data analysis

Three each independent samples of the wild type and mutant strains were inoculated in 50 ml YPD broth and cultured overnight at 30°C in a shaking incubator. In 100 ml fresh YPD broth with an OD_{600} of 0.2, the cells were subcultured until the OD_{600} reached 0.6 or 0.8 at 30°C or 40°C, respectively. Each culture was washed three times with PBS and placed in liquid nitrogen. Total RNA was isolated using the TRIzol reagent (Invitrogen Life Technologies). The concentration, quality, and integrity of total RNA were determined using a NanoDrop spectrophotometer (Thermo Scientific). To prepare an RNA sample, 3 µg RNA was used as input material. Sequencing libraries were constructed using the TruSeq RNA Sample Preparation Kit (Illumina, San Diego, CA, USA). Briefly, mRNA was purified from total RNA using poly-T oligo-attached magnetic beads. Fragmentation was carried out at an elevated temperature using divalent cations in an Illumina proprietary fragmentation buffer. First-strand cDNA was synthesized using random oligonucleotides and SuperScript II. Second-strand cDNA synthesis was subsequently performed using DNA Polymerase I and RNase H. Remaining overhangs were converted into blunt ends via exonuclease/polymerase activities, and the enzymes were removed. After adenylation of the 3′ ends of the DNA fragments, Illumina PE adapter oligonucleotides were ligated to

prepare for hybridization. To select cDNA fragments of the preferred 200-bp length, the library fragments were purified using the AMPure XP system (Beckman Coulter, Beverly, CA, USA). DNA fragments with ligated adaptor molecules on both ends were selectively enriched using an Illumina PCR Primer Cocktail in a 15-cycle PCR reaction. Products were purified (AMPure XP system) and quantified using an Agilent high-sensitivity DNA assay and an Agilent 2100 Bioanalyzer. The sequencing library was then sequenced on a Hiseq platform (Illumina) by Shanghai Personal Biotechnology Cp. Ltd. The Cryptococcus_neoformans_var._grubii_H99 reference genome and gene annotation set were retrieved from Ensemble for alignment. Differentially expressed genes were detected using the Bioconductor package DESeq2 version 1.22.2 [2].

Bioinformatics methods

Gene ontology was performed using R 3.4.3, clusterprofiler 3.6.0, and Annotation Hub (2.12.1) as described previously [3]. *C. neoformans* gene ontology data were downloaded from Annotation Hub, and KOBAS 3.0 was employed to perform KEGG analyses for *C. neoformans* genes. Principal component analysis (PCA) was performed using the devtools and ggbiplot packages in R 3.4.3, whereas a heat map diagram was generated using the pheatmap package, and a correlation analysis was performed using the

corrplot package. A Venn diagram was constructed using the Venny 2.1 website (https://bioinfogp.cnb.csic.es/tools/venny), then NLS and MTS were predicted using NLS Mapper (http://nls-mapper.iab.keio.ac.jp/cgi-bin/NLS_Mapper_form.cgi) and Mitoprot (https://ihg.helmholtz-muenchen.de/ihg/mitoprot.html), respectively. Conservation between *Cn*Hsf3 and hHsf1 (PDB 2ldu.1.A) was calculated using Consurf (consurf.tau.ac.il), and the mitochondrial complexes I, III, and IV in the electron transport chain were modeled using PyMol.

Protein expression and purification in vitro

The DNA sequence encoding *Cn*HSF3-part1 or *Cn*HSF3-part2 was cloned, using the GXD326/GXD446 or GXD447/GXD448 primer pair, respectively, into the *pET30a* expression plasmid containing the 6xHis tag. The cloned plasmid was transformed into BL21(DE3) E. coli, which expressed *Cn*Hsf3-part1 or *Cn*Hsf3-part2. Each was cultured overnight in TB media (Terrific Broth Medium), supplemented with 50 µg/ml kanamycin, then subcultured in a 1-L conical flask containing 1 L TB with 50 µg/ml kanamycin. The culture was grown until an OD600 of 0.6 to 1 was reached, then induced with 0.4 mM IPTG for 16 hours at 16°C.

Cell lysis was achieved by adding 5 mM MgSO4 to the cells for osmotic shock, then centrifuging at 4000 g for 40 minutes at 4°C. Pellets were resuspended in lysis buffer (50 mM Tris-HCl) with 1 mM PMSF, a protease inhibitor cocktail (CWBIO CW2200S), 1 mg/mL lysozyme, and 0.1 mg/g DNaseI, then incubated while stirred at 4°C for 1 hour. The lysates were spun down at 4000 g for 1 hour at 4°C.

To purify the *Cn*Hsf3-part1 protein, the lysates were incubated by adding Ni-NTA at a rate of 3 ml/min at 4°C then washing with washing buffer (50 mM HEPES, 60 mM imidazole, and 200 mM NaCl) and eluted with elution buffer (50 mM HEPES, 150 mM imidazole, and 200 mM NaCl). The final solution was concentrated in a centrifuge tube (30KD, 20 ml) and processed using a SEC650 molecular sieve.

To purify the *Cn*Hsf3-part2 protein, cells were spun down and resuspended in buffer I (50 mM Tris-HCl, 0.5% Triton X-100, 100 mM NaCl, 1 mM NaEDTA, and 1 mM DTT), then harvested at 1000 g for 2 minutes at 4°C. They were resuspended in buffer II (50 mM Tris-HCl, 100 mM NaCl, 1 mM NaEDTA, and 1 mM DTT), then spun down again at 1000 g for 2 minutes at 4°C. After dissolving in a denaturation solution containing 6M guanidine hydrochloride and 4 mM DTT, the resuspended pellet was agitated overnight at 4°C, then

mixed with renaturation buffer (100 mM Tris-HCl, 400 mM L-arginine, 2 mM NaEDTA, 0.5 mM oxidized glutathione, and 5 mM reduced glutathione) with protease inhibitors. After renaturing, the protein solution was filtered using a 2 µm filter and concentrated in a centrifuge tube (30KD, 20 ml). The final solution was centrifuged at 4,000 g for 10 minutes, and the supernatant was processed through a SEC650 molecular sieve to capture the purified *Cn*Hsf3-part2 protein.

To detect oxidation of the *Cn*Hsf3-part1 protein, 20 µg purified *Cn*Hsf3-part1 protein was treated with NaClO at the indicated molar excess for 60 minutes at room temperature, then detected with or without 1% 2-mercaptoethanol using Western blot analysis. Immunoblotting assays were performed using anti-His mouse monoclonal antibody (1:5000 dilution; Sigma) and goat anti-mouse IgG (H+L) HRP secondary antibody (1:5000 dilution; Thermo Fisher Scientific) antibodies. The signal was captured using a ChemiDoc XRS+ (Bio-Rad).

Metabolomic profiling and data processing

Independently, six samples each of wild type and *Cnhsf3* deletion strain were grown in YPD medium overnight and subcultured in 50 ml YPD medium with an OD600 of 0.2 until the OD600 reached 0.6 to 0.8 at 40°C in a shaking

incubator. Cells were washed twice with ice-cold PBS. Samples were thawed on ice, then 500 μL pre-cooled extractant (80% methanol aqueous solution) was added and whirled for 2 minutes. The mixture was subjected to 3 liquid nitrogen freeze-thaw cycles (5 minutes in liquid nitrogen and 5 minutes on ice), then the mixture was centrifuged at 5,000 g at 4°C for 20 minutes. Finally, the supernatant was added to a sample bottle for LC-MS/MS analysis. The sample extracts were analyzed using an LC-ESI-MS/MS system (UPLC, Shim-pack UFLC SHIMADZU CBM30A system, https://www.shimadzu.com/; MS, QTRAP® System, https://sciex.com/). The analytical conditions were as follows: UPLC column, SeQuant ZIC-pHILIC 5 μm (2.1 × 100 mm); column temperature, 40°C; flow rate, 0.4 mL/min; injection volume, 2 μL; solvent system, 10 mmol/L ammonium acetate +0.3% ammonia solution, 90% acetonitrile-water; gradient program, 5:95 V/V at 0 min, 50:50 V/V at 9.5 min, 5:95 V/V at 11.1 min, and 5:95 V/V at 14.0 min. LIT and triple quadrupole (QQQ) scans were acquired using a triple quadrupole-linear ion trap mass spectrometer (QTRAP) and a QTRAP® LC-MS/MS System equipped with an ESI turbo ion-spray interface operating in positive and negative ion modes and controlled by Analyst 1.6.3 software (Sciex). The ESI source operating parameters were as follows: source temperature, 450°C; ion spray (IS) voltage, 5500 V (positive) and -4500 V (negative); collision gas setting (CAD), medium; and gas pressures of 40, 55, and 35.0 psi for the ion source gas I

(GSI), gas II (GSII), and curtain gas (CUR), respectively. Instrument tuning and mass calibration were performed using 10 and 100 μmol/L polypropylene glycol solutions in QQQ and LIT modes, respectively. A specific set of MRM transitions were monitored for each period based on the metabolites eluted within that period. Mass spectral data were processed using Analyst 1.6.3. Based on the local energy metabolism database, the characteristic ions of each substance were screened out by triple quadrupole, and MultiQuant software was used to integrate and calibrate the chromatographic peaks. The peak area of each chromatographic peak represents the relative content of the corresponding substance. Metabolites differing between groups using Two-tailed unpaired t-tests by were found to be significant when $p<0.05$.

2. Supplementary Figures

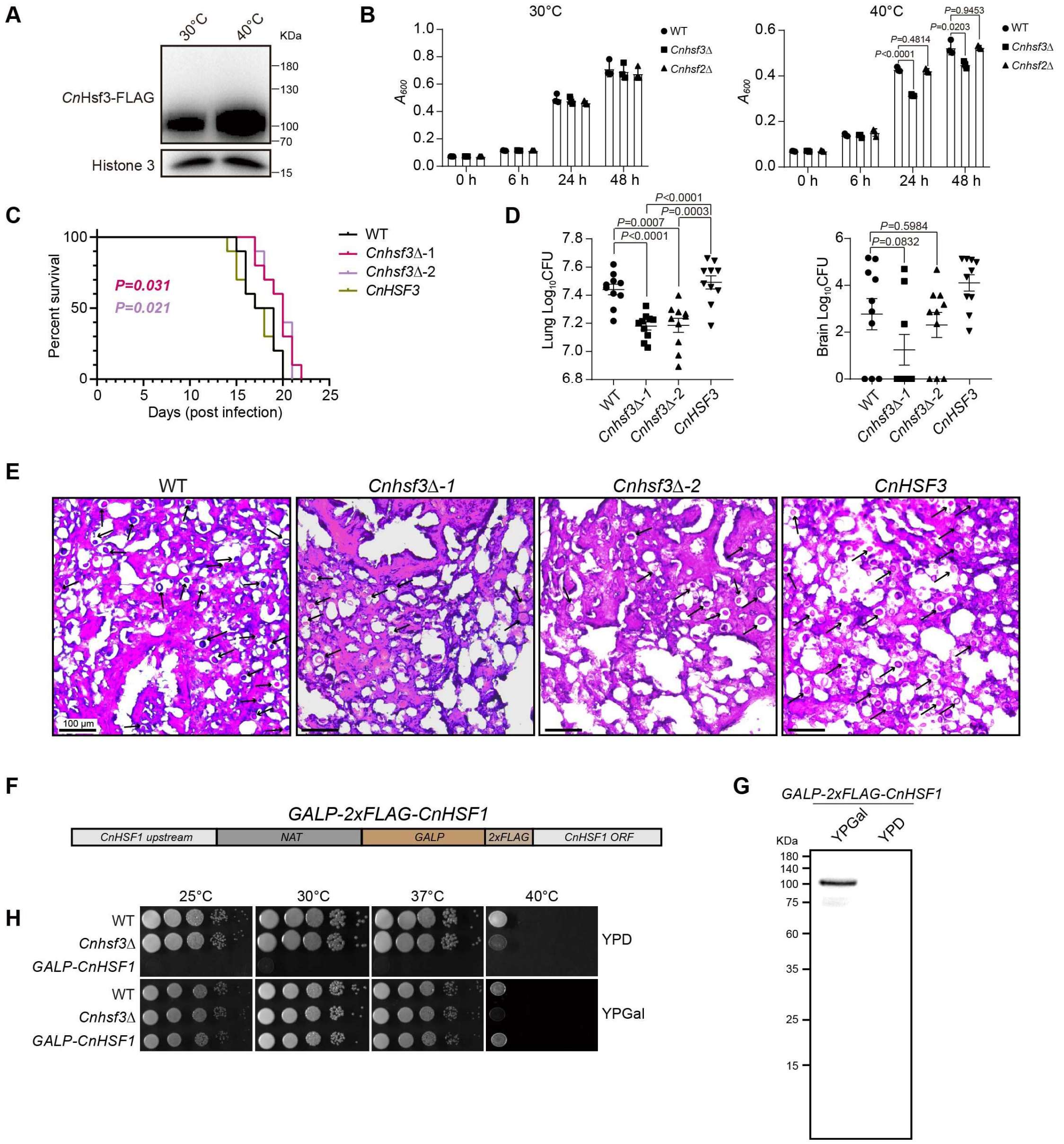

Supplementary Figure 1. *Cn*Hsf3 is a heat shock and fungal pathogenicity regulator.

A. Immunoblotting analysis of *Cn*Hsf3-FLAG. Protein samples were isolated from cells grown at 30°C or 40°C. The immunoblotting of *Cn*Hsf3-FLAG was performed using anti-FLAG antibody. Histone 3 was used as a loading control. Data were obtained from three independent experiments with and representative images are shown.

B. Quantification of fungal growth in liquid cultures. Indicated strain cells (n=3) were grown in YPD liquid media at 30°C or 40°C. Cell growths (absorbance at 600 nm) were measured at indicated time points. P values were calculated using two-tailed unpaired t-tests. Data are expressed as mean ± SD.

C. Animal survival analysis and the Kaplan-Meier survival plot of wildtype, *Cnhsf3Δ* (two independent knockout strains), and *CnHSF3* (complementation strain) (n=10). Significance was determined using Log-rank (Mantel-Cox) test.

D. CFUs analysis of infected lung and brain tissues. CFUs were enumerated after 14 days of infection. CFUs were normalized to tissue weight (n=10). P values were calculated using two-tailed unpaired t-tests. Data are expressed as mean ± SEM. Data were obtained from two independent experiments with and representative images are shown.

E. Histopathology analyses of infected lung tissues. Infected lung tissues were strain with Periodic Acid-Schiff (PAS) and examined under a x10 lens (scale bar = 100 μm). Stained fungal cells are indicated with arrows. Data were obtained from three independent experiments with and representative images are shown.

F. Scheme of GAL*P-CnHSF1* strain construction.

G. Immunoblotting analysis of *GALP-CnHSF1*. The *GALP-CnHSF1* strain was grown in YPGal or YPD media. Protein samples were isolated, and immunoblotting was performed using anti-FLAG antibody. Data were obtained from three independent experiments with and representative images are shown.

H. Spotting assay of *Cnhsf3Δ* and *GALP-CnHSF1* strains. Cells were spotted onto YPD or YPGal agar plates, which were incubated at indicated temperatures for 3 days.

Source data are provided as a Source Data.

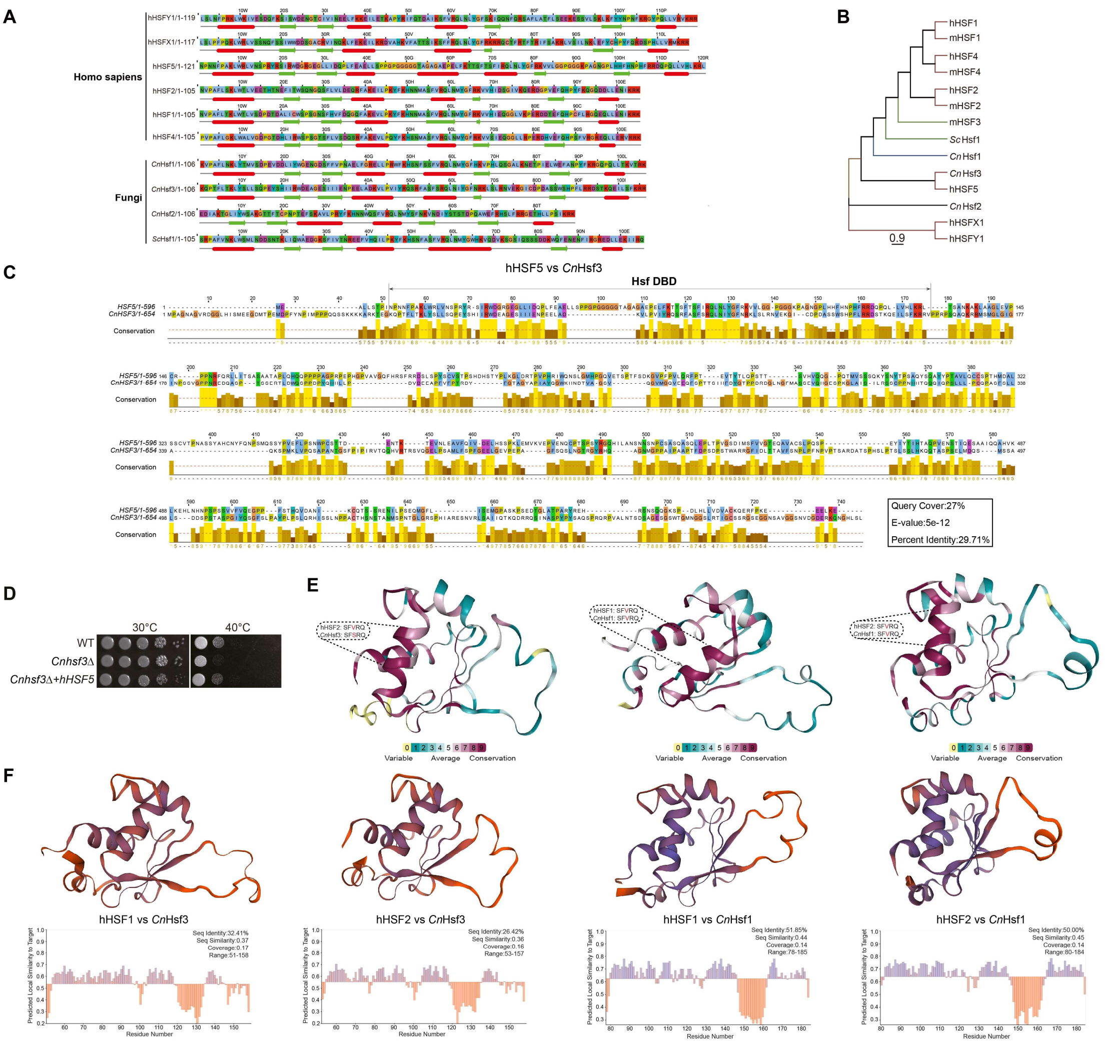

Supplementary Figure 2. *Cn*Hsf3 is homologous to HSFs from other species.

A. Human and fungal HSF DBDs were aligned, and secondary structures were analyzed using Clustal Omega software.

B. Phylogenetic analysis of HSFs.

C. Residue conversation between the DBD structures of hHSF5 and *Cn*Hsf3 was performed by Clustal Omega software.

D. Spotting assay of the *Cnhsf3*Δ+hHSF5 strain. Cells were spotted onto YPD agar plates, which were incubated at indicated temperatures for 3 days.

E. Homologous modeling of the DBD comparison including CnHsf3 and hHsf2, CnHsf1 and hHsfF1, CnHsf1 and hHsf2, respectively. The DBD structures of CnHsf1 and CnHsf3 were predicted and compared with that of hHsf1 or hHsf2 using ConSurf software[4, 5].

F. The structural similarity of CnHsf1 and CnHsf3 with hHsf1 and hHsf2. The DBD structures of CnHsf1 and CnHsf3 were mimicked by hHsf1 [PDB: 5d5u (https://swissmodel.expasy.org/repository/uniprot/Q00613?template=5d5u)] and hHsf2 [PDB: 5hdk(https://swissmodel.expasy.org/repository/uniprot/Q03933?template=5hdk)], and the predicted local similarity to target was calculated using SWISS-MODEL (swissmodel.expasy.org)[6].

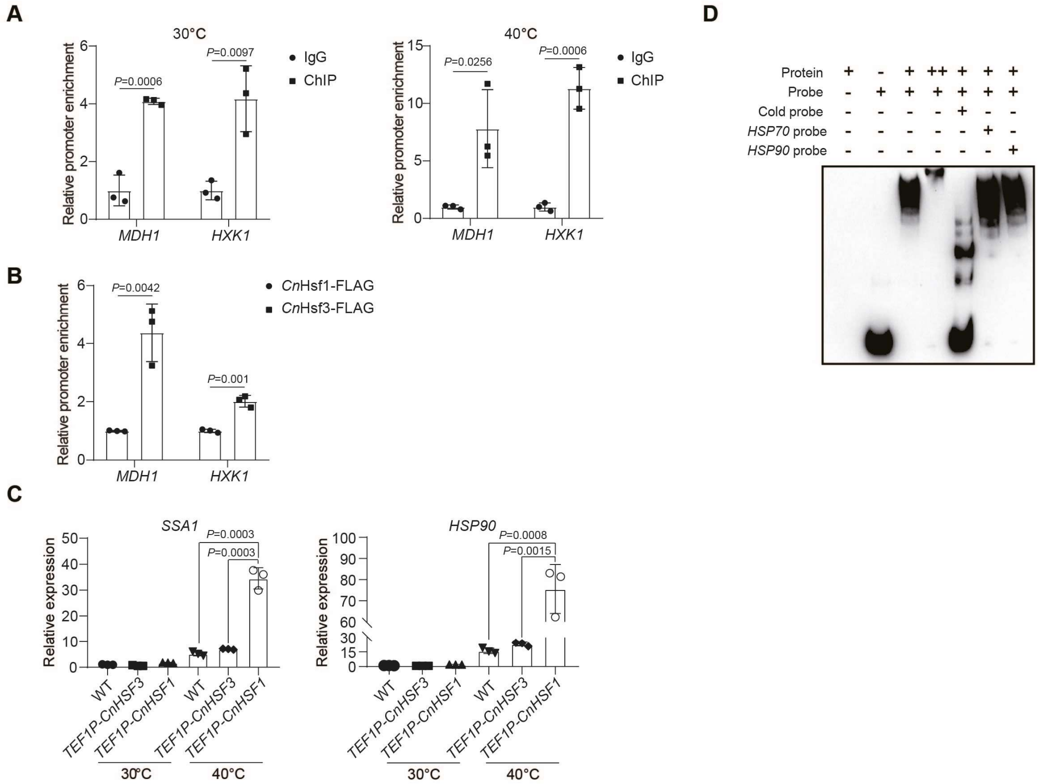

Supplementary Figure 3. *Cn*Hsf3 is an atypical HSF.

A. ChIP-PCR analysis of *Cn*Hsf3-FLAG. ChIP assays were performed in *Cn*Hsf3-FLAG strain (n=3) grown at 30°C or 40°C. Promoter enrichments for *MDH1* and *HXK1* were analyzed using PCR.

B. ChIP-PCR analysis of *Cn*Hsf3-FLAG and *Cn*Hsf1-FLAG. ChIP assays were performed in *Cn*Hsf3-FLAG and *Cn*Hsf1-FLAG strains (n=3). Promoter enrichments for *MDH1* and *HXK1* were analyzed using PCR.

C. qRT-PCR analysis of gene expression of *SSA1* and *HSF90*. Indicated strains (n=3) were grown 30°C or 40°C. Gene expression of *SSA1* and *HSF90* were analyzed using qRT-PCR.

D. EMSA analysis of CnHsf3 binding to HSF promoters. Purified CnHsf3 DBD domain was incubated with probe (biotin-labeled CnHsf3 target DNA sequence), cold probe (unlabeled CnHsf3 target DNA sequence), *HSP70* promoter sequence or *HSP90* promoter sequence. Data were obtained

from two independent experiments with and representative images are shown.

Data are expressed as mean ± SD. P values were calculated using two-tailed unpaired t-tests. Source data are provided as a Source Data.

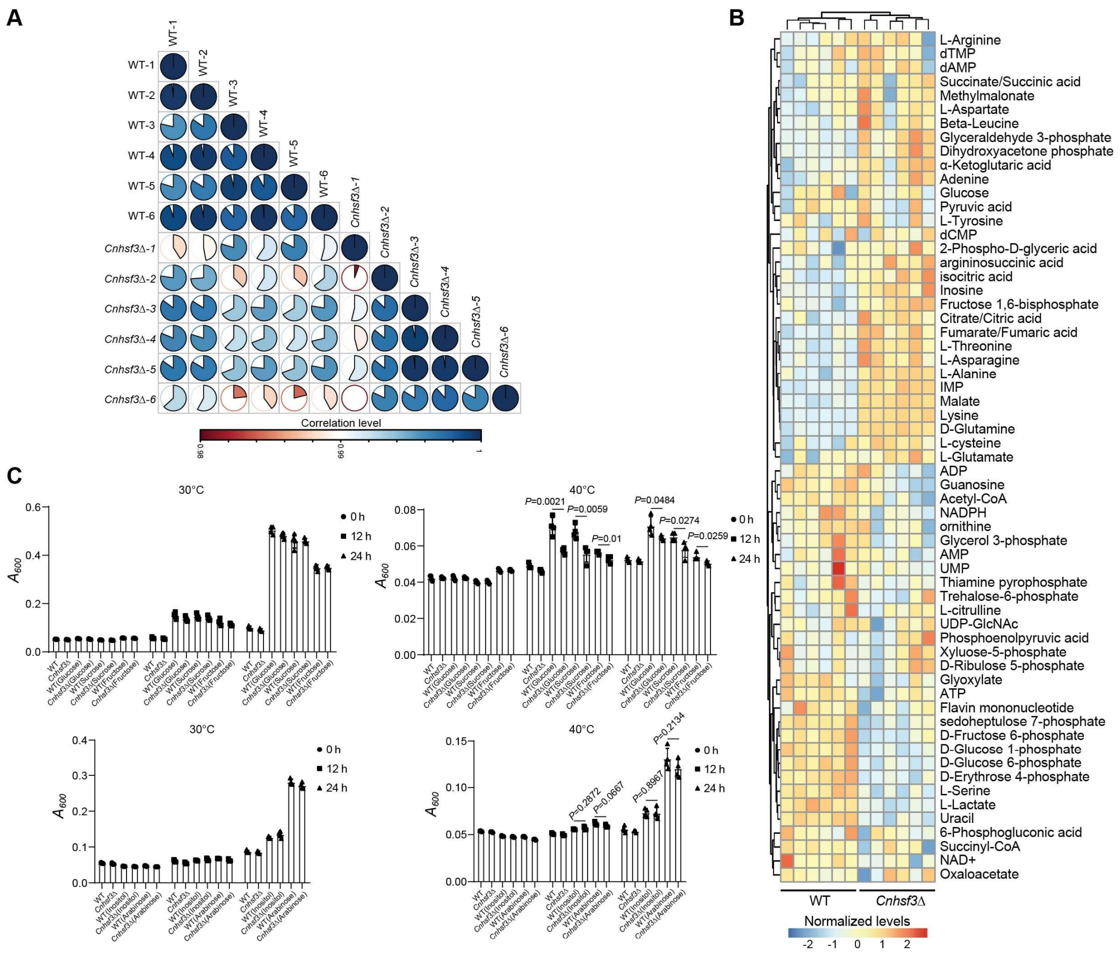

Supplementary Figure 4. Metabolome analyses in wildtype and *Cnhsf3Δ* strains.

A. Correlation analysis between the metabolomes of wild type (*n*=6) and *Cnhsf3Δ* (*n*=6) strains. The correlation was determined using the Corrplot package, version 0.84

B. The metabolome data were used to generate heat maps for wild type and *Cnhsf3Δ* strains. Normalized levels indicate concentrations of detected metabolites.

C. Quantification of fungal growth in various carbon liquid media. Wildtype and *Cnhsf3Δ* cells (n=4) were grown in YNB liquid media supplemented with the carbon sources indicated. The control was provided no sugar supplement.

Data are expressed as mean ± SD. P values were calculated using two-tailed unpaired t-tests. Source data are provided as a Source Data.

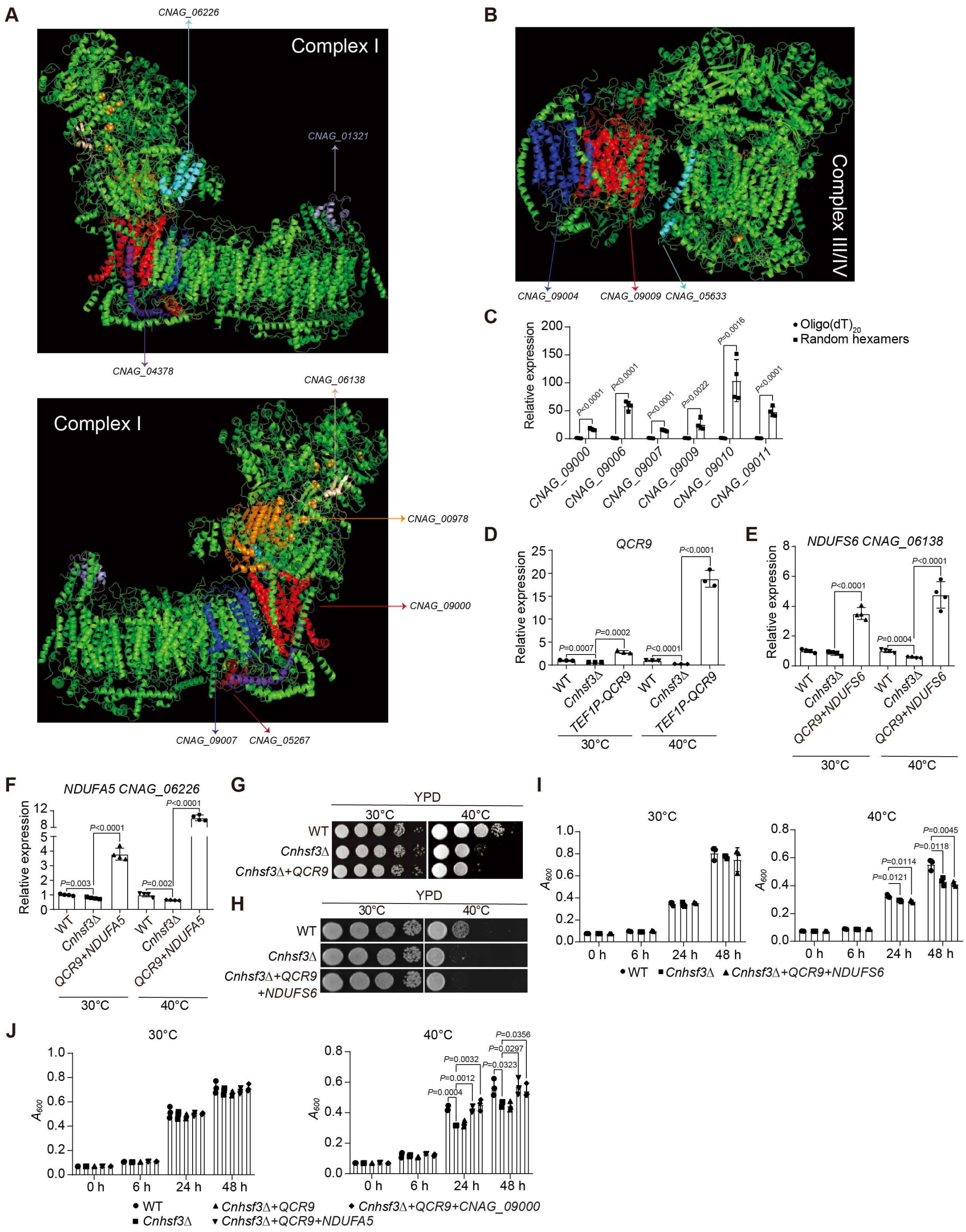

Supplementary Figure 5. *Cn*Hsf3 regulates gene expression of respiration complexes.

A-B. *Cn*Hsf3 regulates gene encoding of the subunits of complexes I, III, and IV. Protein structures [6GCS (https://www.rcsb.org/structure/6GCS) and

6YMX (https://www.rcsb.org/structure/6YMX)] were downloaded from the Protein Data Bank, then *Cn*Hsf3-regulated gene products were mapped.

C. RT-PCR analysis of mitochondrial encoding genes using oligo dT or random hexamers (n=4).

D. qRT-PCR was used to quantify *QCR9* expression in cells (*n*=3) harboring plasmid *TEF1-QCR9*.

E-F. qRT-PCR was used to quantify *NDUFS6* and *NDUFA5* overexpression in cells (*n*=4).

G-H. *QCR9*- and *QCR9*+*NDUFS6*-overexpressing cells were spotted onto YPD agar and incubated at 30°C or 40°C for 2 days to provide the spotting assay.

I-J. Quantification of fungal growth in liquid cultures. Indicated strain cells (n=3) were grown in YPD liquid media at 30°C or 40°C. Cell growths (absorbance at 600 nm) were measured at indicated time points.

Data are expressed as mean ± SD. P values were calculated using two-tailed unpaired t-tests. Source data are provided as a Source Data.

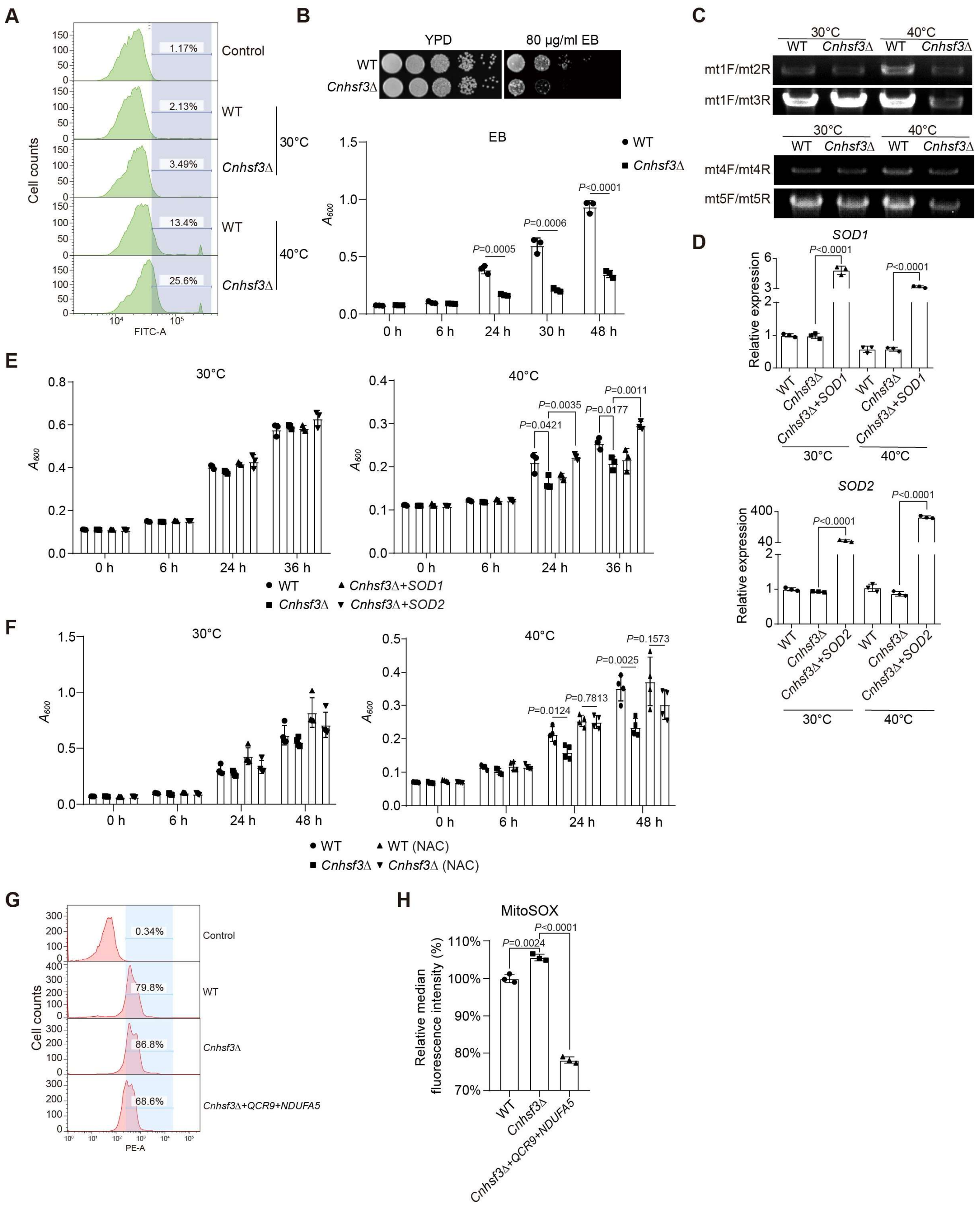

Supplementary Figure 6. *Cn*Hsf3 balances mitochondrial ROS levels.

A. Flow cytometry was used to analyze H2DCFDA-stained cells that, once loaded, were grown at 30°C or 40°C for 30 minutes.

B. Wildtype and *Cnhsf3Δ* cells were spotted onto YPD agar or liquid culture with or without 80 μg/ml ethidium bromide, and plates were incubated at

30°C for 3 days. Cell (n=3) densities were measured at indicated time points.

C. PCR was used to test mitochondrial genome integrity, employing four pairs of oligos (mt1F/mt2R, mt1F/mt3R, mt4F/mt4R, and mt5F/mt5R) in wildtype and *Cnhsf3Δ* cells grown for 30 minutes at 30°C or 40°C.

D. Expression of *SOD* was quantified using qRT-PCR on *Cnhsf3Δ* cells transformed with overexpressing plasmids harboring *SOD1* or *SOD2* (*n*=3).

E. Quantification of fungal growth in liquid cultures. Indicated strain cells (n=3) were grown in YPD liquid media at 30°C or 40°C. Cell growths (absorbance at 600 nm) were measured at indicated time points.、

F. Quantification of fungal growth in liquid cultures. Indicated strain cells (n=4) were grown in YPD liquid media supplemented with or without 3 mM NAC at 30°C or 40°C. Cell growths (absorbance at 600 nm) were measured at indicated time points.

G-H. Flow cytometry was used to analyze MitoSOX-stained cells (n=3) that, once loaded, were grown at 40°C for 30 minutes.

Data are expressed as mean ± SD. P values were calculated using two-tailed unpaired t-tests. Source data are provided as a Source Data.

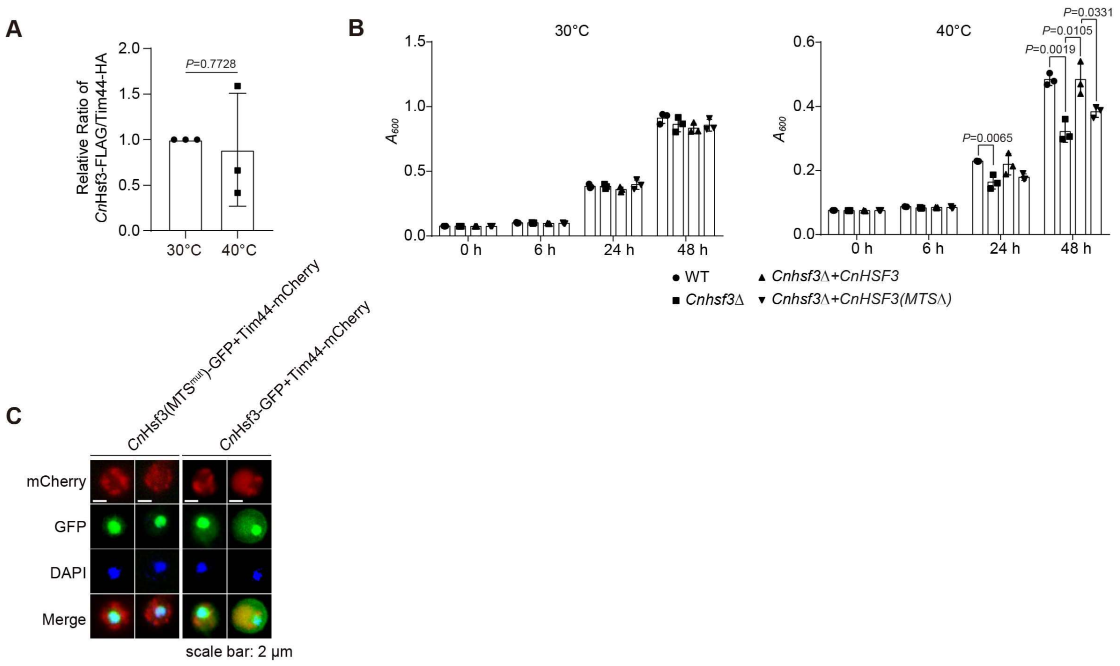

Supplementary Figure 7. *Cn*Hsf3 is a nuclear and mitochondrial localized transcription factor.

A. Relative protein ratio of *Cn*Hsf3-FLAG and Tim44-HA. Relative ratio of mitochondrial *Cn*Hsf3-FLAG (n=3) was calculated from Figure 4C and S10E. The signal intensities of Tim44-HA were used as controls

B. Quantification of fungal growth in liquid cultures. Indicated strain cells (n=3) were grown in YPD liquid media at 30°C or 40°C. Cell growths (absorbance at 600 nm) were measured at indicated time points.

C. Localization of *Cn*Hsf3-GFP. Cells expressing Tim44-mCherry and *Cn*Hsf3 (MTS^{mut})-GFP or *Cn*Hsf3-GFP were analyzed using a fluorescence microscope. Nuclei were stained with DAPI. Scale bars = 2 microns. Data were obtained from three independent experiments with and representative images are shown.

Source data are provided as a Source Data.

Data are expressed as mean ± SD. P values were calculated using two-tailed unpaired t-tests.

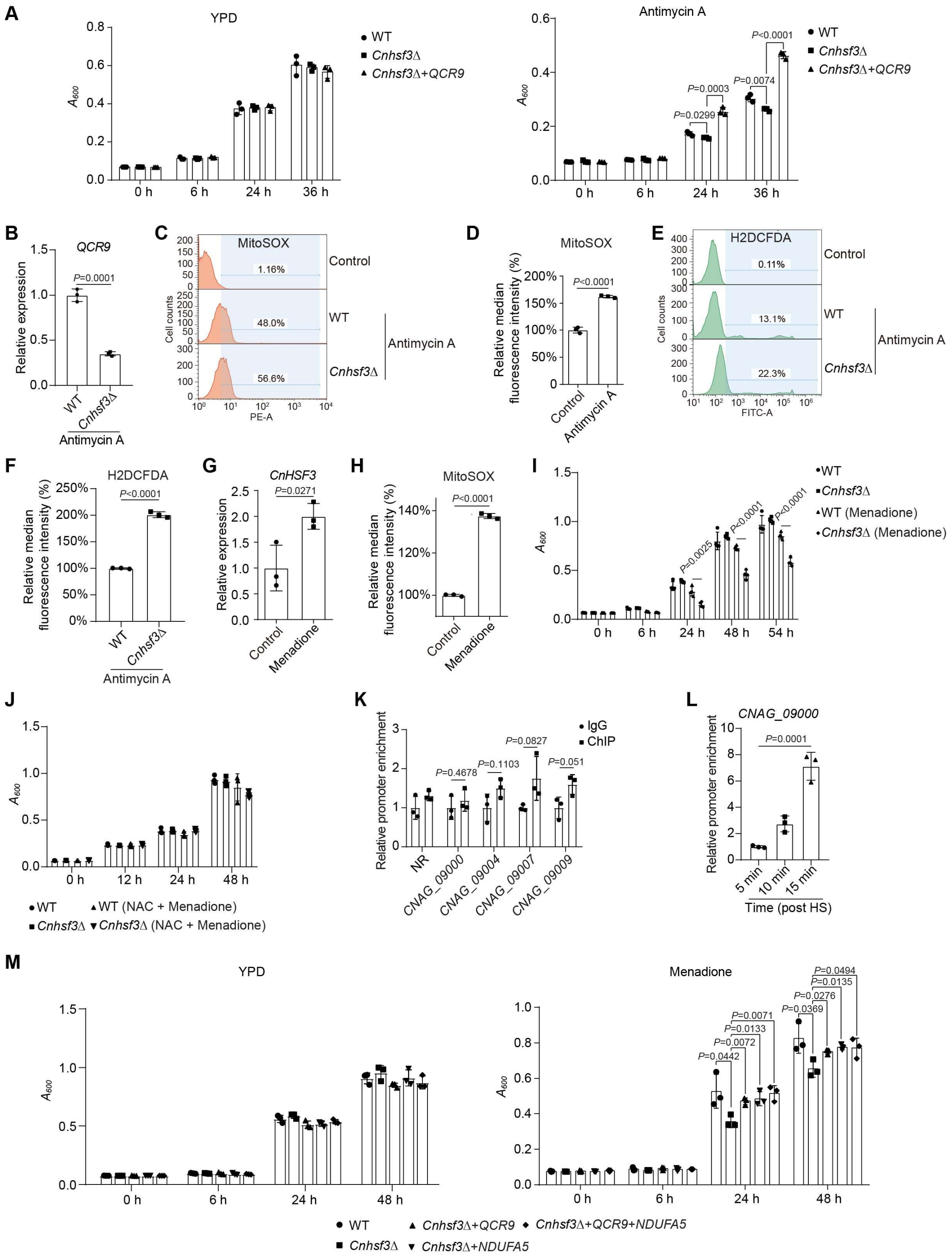

Supplementary Figure 8. *Cn*Hsf3 is a detoxifying agent in mitochondria.

A. Fungal growth. Strains (n=3) were grown in YPD liquid media with antimycin A (10 μM) at 30°C. Cell growths were measured. Two-tailed unpaired t-tests were used.

B. Gene expression of *QCR9* in response to antimycin A was measured in wildtype and *Cnhsf3Δ* (n=3). Two-tailed unpaired t-tests were used.

C. Cell population analysis of MitoSOX. Strains were incubated with antimycin A at 30°C. Flow cytometry was performed.

D. Quantification of MitoSOX. Fluorescence signals were quantified and plotted (n=3). Two-tailed unpaired t-tests were used.

E. Cell population analysis of H2DCFDA. Indicated fungal strains were incubated in the presence of antimycin A at 30°C, then H2DCFDA -based flow cytometry was performed.

F. Quantification of H2DCFDA. Fluorescence signals were quantified and plotted (n=3). Two-tailed unpaired t-tests were used.

G. Wildtype cells (*n*=3) were treated with 30 μM menadione at 30°C for 3 hours, then qRT-PCR was used to determine the expression of *CnHSF3*. Two-tailed unpaired t-tests were used.

H. MitoSOX staining and flow cytometry were used to quantify mtROSs in wildtype cells (*n*=3) treated with menadione, then incubated at 30°C for 30 minutes. Two-tailed unpaired t-tests were used.

I-J. Quantification of growth in liquid cultures. Strains (n=3) were grown in YPD liquid media supplemented with 30 μM menadione or 30 μM menadione and 1.5 mM NAC at 30°C. Growths were measured at indicated time points. Two-tailed unpaired t-tests were used.

K. The *CnHSF3-FLAG* strain (*n*=4) was grown at 30°C (normal growth conditions) for 3 hours. ChIP-PCR was performed. Two-tailed unpaired t-tests were used.

L. *Cn*Hsf3-FLAG ChIP-PCRs of *CNAG_09000*. The *CnHSF3-FLAG* strain (n=3) was incubated at 40°C for 5 min, 10 min and 15 min. ChIPs were performed, oligos for *CNAG_09000* promoter were used. One-way ANOVA test was used.

M. Quantification of fungal growth in liquid cultures. Indicated strains (n=3) were grown in YPD liquid media supplemented with 30 μM menadione at 30°C. Cell growths were measured at indicated time points. Two-tailed unpaired t-tests were used.

Data are expressed as mean ± SD. Source data are provided as a Source Data.

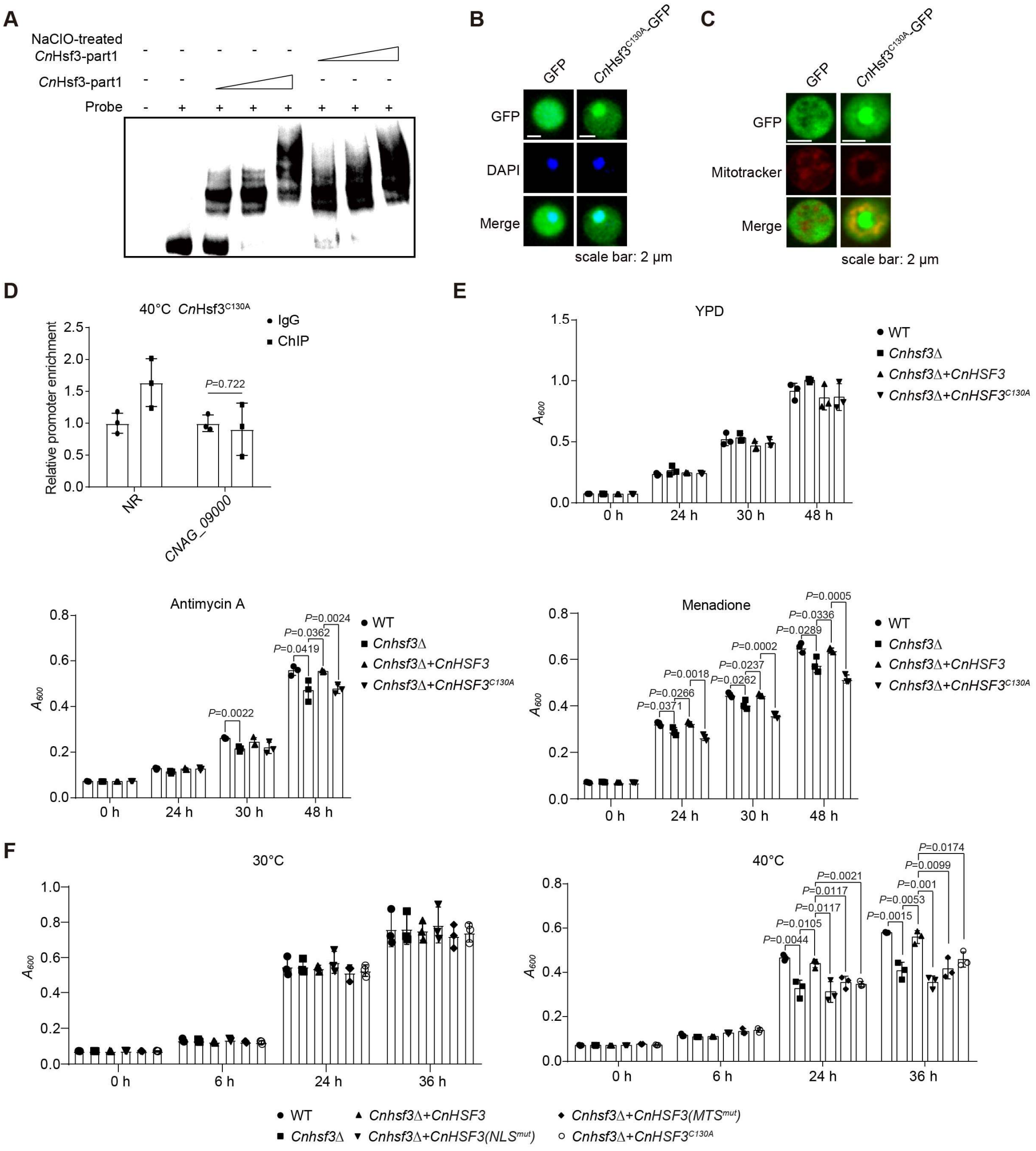

Supplementary Figure 9 Cysteine 130 oxidation of *Cn*Hsf3 is critical for protein function.

A. EMSA analysis of *Cn*Hsf3 DBD. *Cn*Hsf3 DBD (*Cn*Hsf3-part1) was expressed and purified. EMSA assay was performed using *Cn*Hsf3-part1 and NaClO-treated *Cn*Hsf3-part1. Data were obtained from three independent experiments with and representative images are shown.

B-C. Localization of *Cn*Hsf3^{C130A}-GFP. Cells expressing GFP or *Cn*Hsf3^{C130A}-GFP were analyzed using a fluorescence microscope. Nuclei were stained with DAPI, and mitochondria were stained with Mitotracker. Scale

bars = 2 microns. Data were obtained from three independent experiments with and representative images are shown.

D. *Cn*Hsf3^{C130A}-FLAG ChIP-PCR analysis. The *CnHSF3* ^{C130A} *-FLAG* strain (n=3) was incubated at 40°C. ChIP assays were performed. PCRs were carried out using oligos for *CNAG_09000* promoter and NR (unregulated region) for negative control.

E. Quantification of fungal growth in liquid cultures. Indicated strain cells (n=3) were grown in YPD liquid media supplemented with antimycin A or menadione at 30°C. Cell growths (absorbance at 600 nm) were measured at indicated time points.

F. Quantification of fungal growth in liquid cultures. Indicated strain cells (n=3) were grown in YPD liquid media at 30°C or 40°C. Cell growths (absorbance at 600 nm) were measured at indicated time points.

Data are expressed as mean ± SD. P values were calculated using two-tailed unpaired t-tests. Source data are provided as a Source Data.

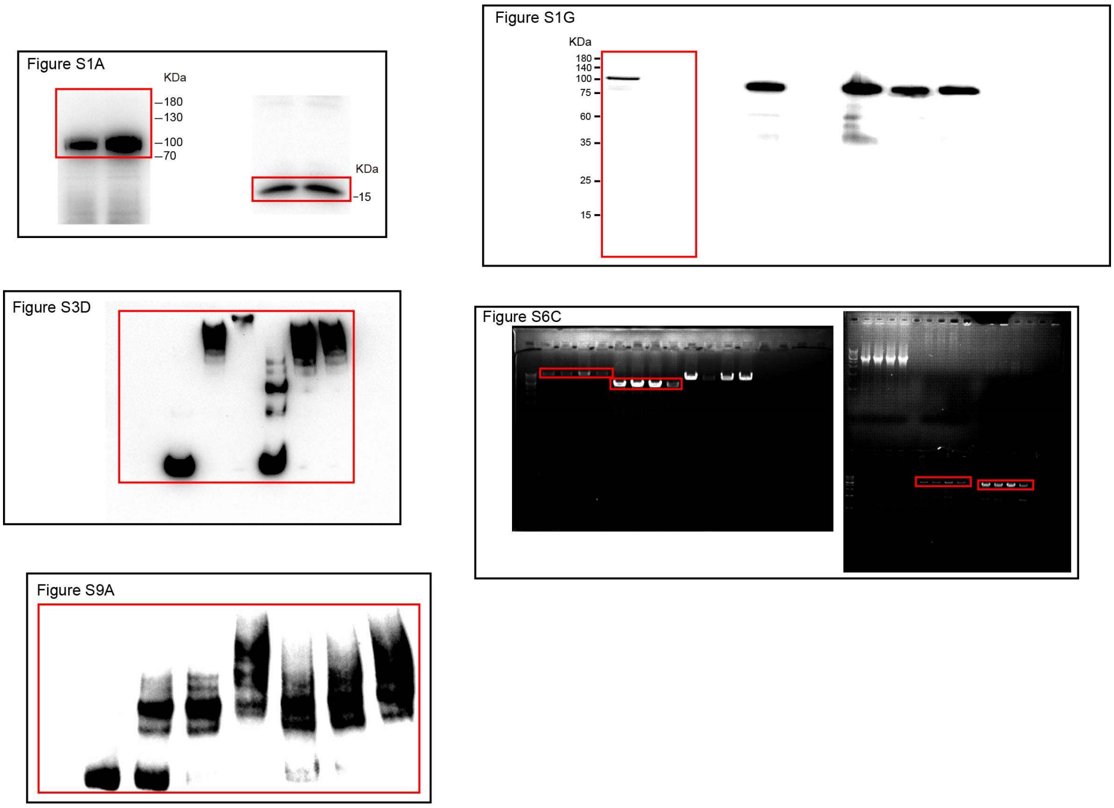

Supplementary Figure 10 Immunoblotting raw data in Supplementary Information

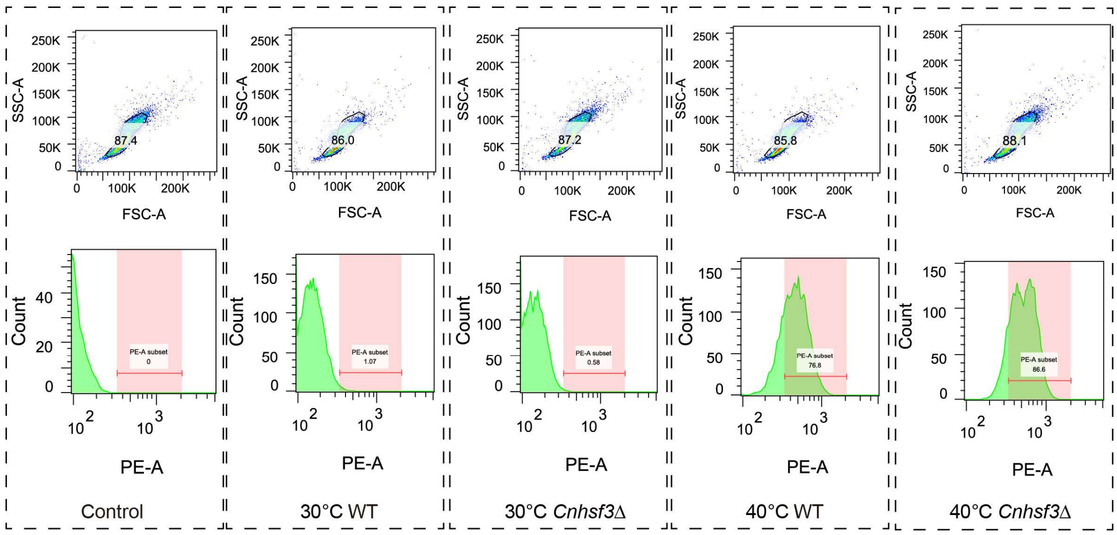

Supplementary Figure 11 Representative flow cytometry gating

Unstained cells were used as the control group. The fluorescence intensity of cells was higher than the control group as the positive cell population. The cell population with the highest cell density was analyzed for the average fluorescence intensity, and the same FSC/SSC parameter was used for each group.

3. Supplementary References

1. Toffaletti, D.L., Rude, T.H., Johnston, S.A., Durack, D.T. & Perfect, J.R. Gene transfer in Cryptococcus neoformans by use of biolistic delivery of DNA. *J Bacteriol* **175**, 1405-1411 (1993).
2. Anders, S. & Huber, W. Differential expression analysis for sequence count data. *Genome Biol* **11**, R106 (2010).
3. Huber, W. *et al.* Orchestrating high-throughput genomic analysis with Bioconductor. *Nat Methods* **12**, 115-121 (2015).
4. Ashkenazy, H. *et al.* ConSurf 2016: an improved methodology to estimate and visualize evolutionary conservation in macromolecules. *Nucleic Acids Res* **44**, W344-350 (2016).

5. Jaeger, A.M., Pemble, C.W.t., Sistonen, L. & Thiele, D.J. Structures of HSF2 reveal mechanisms for differential regulation of human heat-shock factors. *Nat Struct Mol Biol* **23**, 147-154 (2016).
6. Waterhouse, A. *et al.* SWISS-MODEL: homology modelling of protein structures and complexes. *Nucleic Acids Res* **46**, W296-W303 (2018).
