# Supplementary material for: Cryptococcal Hsf3 controls intramitochondrial ROS homeostasis by regulating the respiratory process
Source: Nat Commun. 2022 Sep 15;13:5407. doi: 10.1038/s41467-022-33168-1 (PMC9477856; doi:10.1038/s41467-022-33168-1)
Supplement: Supplementary file 3 — Description of Additional Supplementary Files [file 41467_2022_33168_MOESM3_ESM.pdf]

Supplementary Data 1: ChIP-seq analysis in the CnHsf1-FLAG and CnHsf3-FLAG strain.

Supplementary Data 2: Transcriptome analysis in C. neoformans knock-outs or wild-type cells at indicated temperature.

Supplementary Data 3: GO and KEGG analysis of DEG (differential expressed genes) in C. neoformans knock-outs or wild-type cells treated at indicated temperature and GO analysis of genes of CnHsf1-Flag or CnHsf3-Flag ChIP-seq in C. neoformans at 40 °C.

Supplementary Data 4: Metabolomic analysis in C. neoformans knock-outs or wild-type cells at 40°C.

Supplementary Data 5: Mass spectrometry analysis of CnHsf3-interacting proteins.

Supplementary Data 6: Primers used in this study.

Supplementary Data 7: Strains used in this study.
