# Supplementary material for: Cryptococcal Hsf3 controls intramitochondrial ROS homeostasis by regulating the respiratory process
Source: Nat Commun. 2022 Sep 15;13:5407. doi: 10.1038/s41467-022-33168-1 (PMC9477856; doi:10.1038/s41467-022-33168-1)
Supplement: Supplementary file 11 — Reporting Summary [file 41467_2022_33168_MOESM11_ESM.pdf]

nature portfolio

Corresponding author(s): Chen Ding

Last updated by author(s): Aug 30, 2022

Reporting Summary

Nature Portfolio wishes to improve the reproducibility of the work that we publish. This form provides structure for consistency and transparency in reporting. For further information on Nature Portfolio policies, see our Editorial Policies and the Editorial Policy Checklist.

Statistics

For all statistical analyses, confirm that the following items are present in the figure legend, table legend, main text, or Methods section.

n/a	Confirmed	
☐	☒	The exact sample size (n) for each experimental group/condition, given as a discrete number and unit of measurement
☐	☒	A statement on whether measurements were taken from distinct samples or whether the same sample was measured repeatedly
☐	☒	The statistical test(s) used AND whether they are one- or two-sided<br>*Only common tests should be described solely by name; describe more complex techniques in the Methods section.*
☒	☐	A description of all covariates tested
☒	☐	A description of any assumptions or corrections, such as tests of normality and adjustment for multiple comparisons
☐	☒	A full description of the statistical parameters including central tendency (e.g. means) or other basic estimates (e.g. regression coefficient) AND variation (e.g. standard deviation) or associated estimates of uncertainty (e.g. confidence intervals)
☒	☐	For null hypothesis testing, the test statistic (e.g. F, t, r) with confidence intervals, effect sizes, degrees of freedom and P value noted<br>*Give P values as exact values whenever suitable.*
☒	☐	For Bayesian analysis, information on the choice of priors and Markov chain Monte Carlo settings
☒	☐	For hierarchical and complex designs, identification of the appropriate level for tests and full reporting of outcomes
☒	☐	Estimates of effect sizes (e.g. Cohen's d, Pearson's r), indicating how they were calculated

Our web collection on statistics for biologists contains articles on many of the points above.

Software and code

Policy information about availability of computer code

Data collection	Data were collected as described in the manuscript.<br>CFX96 real-time system (Bio-Rad) was used to acquire the Real-time PCR data.<br>ChemiDoc XRS+ (Bio-Rad) was used to acquire the Western Blot data.<br>Biacore T200 instrument (Becton Dickinson and Company) was used to acquire the SPR data.<br>Leica TCS SP8 (Leica) was used to acquire the confocal images.<br>BD LSRFortessa Cell Analyzer was used to acquire flow cytometry data.<br>Synergy H4 microplate reader (BioTek) was used to acquire the luminescent signal.<br>MultiskanGO microplate reader (Thermo) was used to acquire the Absorbance data.<br>Illumina Hiseq-PE150 platform (Illumina) was used to acquire transcriptome data.<br>Ultra Performance Liquid Chromatograph(Shimpack UFLC SHIMADZU CBM30A) and Tandem mass spectrometry MS/MS(QTRAP) was used to acquire the Metabolomics data.<br>Orbitrap Q-Exactive-plus was used to acquire the Mass spectrometry data.

Data analysis	Statistical data was performed using GraphPad Prism software (GraphPad 9).<br>Differentially expressed genes were detected using the Bioconductor package DESeq2 version 1.22.2.<br>SPR data was analyzed by Biacore T200 Evaluation Software (Cytiva).<br>MiNA toolset in ImageJ was used to analyze the confocal images.<br>Flow cytometry data was analyzed using FlowJo version 10.0.7r2.<br>Analyst 1.6.3 was used to analyze the mass spectrometric data of Metabolomics.<br>The mass spectrum data obtained was retrieved by Mascot(version 2.5.1) and Maxquant (1.5.2.8) software was used to analyze the quantitative calculations of Mass spectrometry.

For manuscripts utilizing custom algorithms or software that are central to the research but not yet described in published literature, software must be made available to editors and reviewers. We strongly encourage code deposition in a community repository (e.g. GitHub). See the Nature Portfolio guidelines for submitting code & software for further information.

Data

Policy information about availability of data

All manuscripts must include a data availability statement. This statement should provide the following information, where applicable:

- Accession codes, unique identifiers, or web links for publicly available datasets
- A description of any restrictions on data availability
- For clinical datasets or third party data, please ensure that the statement adheres to our policy

C. neoformans H99 genome was downloaded from NCBI (https://www.ncbi.nlm.nih.gov/genome/61?genome_assembly_id=52487).
The authors declare that the data supporting the findings of this study are available within the article and its Supplementary Information files.
The mass spectrometry proteomics data have been deposited to the ProteomeXchange Consortium via the PRIDE partner repository with the dataset identifier PXD033799.
The transcriptome (RNA-seq) and ChIP-seq data are deposited in NCBI's Gene Expression Omnibus (GEO) (https://www.ncbi.nlm.nih.gov/geo/) and can be accessed through GEO Series accession ID GEO: GSE183184.
The mass spectrometry raw data associated with this study have been deposited on the open metabolomics database MetaboLights (ID: MTBLS5745).

Field-specific reporting

Please select the one below that is the best fit for your research. If you are not sure, read the appropriate sections before making your selection.

☒ Life sciences ☐ Behavioural & social sciences ☐ Ecological, evolutionary & environmental sciences

For a reference copy of the document with all sections, see nature.com/documents/nr-reporting-summary-flat.pdf

Life sciences study design

All studies must disclose on these points even when the disclosure is negative.

Sample size	No statistical methods were used to predetermine sample size. Sample sizes were estimated based on standards of this field and preliminary experiments. For in vitro experiment, 3 - 10 sample size was used for analysis; For in vivo experiment , 10 mice per group were used. These sample sizes were sufficient to detect meaningful biological difference with good reproducibility.
Data exclusions	The sequencing data was normalized by corresponding softwares or programs, and no experimental data was excluded.
Replication	All results were repeated two or three independent times. Please refer to Figure Legend for the detailed explanation of each experiment.<br>Similar data were obtained in independent experiments.<br>Three biological replicates were done for RNA-seq, ChIP-seq and mass spectrometry proteomics .<br>Six biological replicates were done for Metabolomic profiling.
Randomization	For the animal experiments, mice were randomly allocated to different experimental groups.
Blinding	The data collections or analyses are blinded for all investigators.

Reporting for specific materials, systems and methods

We require information from authors about some types of materials, experimental systems and methods used in many studies. Here, indicate whether each material, system or method listed is relevant to your study. If you are not sure if a list item applies to your research, read the appropriate section before selecting a response.

Materials & experimental systems

n/a	Involved in the study	
	☒	Antibodies
☒		Eukaryotic cell lines
☒		Palaeontology and archaeology
	☒	Animals and other organisms
☒		Human research participants
☒		Clinical data
☒		Dual use research of concern

Methods

n/a	Involved in the study	
	☒	ChIP-seq
	☒	Flow cytometry
☒		MRI-based neuroimaging

Antibodies

Antibodies used

Anti-Histone H3 (D1H2) XP® Rabbit mAb, CST, Cat# 4499S （1:1000 dilution)
Anti-Flag Mouse Monoclonal Antibody, Transgene, Cat# HT201-01 （1:1000 dilution)
Anti-HA (C29F4) Rabbit mAb, CST, Cat# 3724S （1:1000 dilution)
Anti-His Mouse Monoclonal Antibody, sigma, Cat# SAB1305538 （1:1000 dilution)
Goat Anti-Mouse IgG (H+L) Secondary Antibody, HRP, Thermo Fisher, Cat# 31430 （1:1000 dilution)
Goat Anti-Rabbit IgG (H+L) Secondary Antibody, HRP, Thermo Fisher, Cat# 31460 （1:1000 dilution)

Validation

All antibodys used in this study are commercial and used according to the manufacturer's instructions.

Animals and other organisms

Policy information about studies involving animals; ARRIVE guidelines recommended for reporting animal research

Laboratory animals

The mice were routinely maintained in a pathogen-free animal facility at a temperature of 21°C, relative humidity of 50-70%. All animal experiments were carried out in accordance with the regulation in the Guide for the Care and Use of Laboratory Animals issued by the Ministry of Science and Technology of the People's Republic of China. Mice were cared with an alternating 12 h light-dark cycle and unlimited food and water supply.Six- to eight week-old female BALB/c mice per fungal strain were used for survival analysis, fungal burden assay and histopathology analyses. Infected mice were sacrificed using carbon dioxide.

Wild animals

The study did not involve wild animals

Field-collected samples

The study did not involve field-collected animals

Ethics oversight

All animal experiments were carried out under the review and approval of the Research Ethics Committees at National Clinical Research Center for Laboratory Medicine of the First Affiliated Hospital of China Medical University.

Note that full information on the approval of the study protocol must also be provided in the manuscript.

ChIP-seq

Data deposition

☒ Confirm that both raw and final processed data have been deposited in a public database such as GEO.

☒ Confirm that you have deposited or provided access to graph files (e.g. BED files) for the called peaks.

Data access links
May remain private before publication.

To review GEO accession GSE183184:
Go to https://www.ncbi.nlm.nih.gov/geo/query/acc.cgi?acc=GSE183184

Files in database submission

GSM5552555 TF1.Input
GSM5552556 TF1.ChIP.rep1
GSM5552557 TF1.ChIP.rep2
GSM5552558 TF1.ChIP.rep3
GSM5552559 TX1.Input
GSM5552560 TX1.ChIP.rep1
GSM5552561 TX1.ChIP.rep2
GSM5552562 TX1.ChIP.rep3

Genome browser session
(e.g. UCSC)

http://fungidb.org/fungidb/

Methodology

Replicates	Three replicates:TF1.ChIP.rep1, TF1.ChIP.rep2,TF1.ChIP.rep3 and Three replicates: TX1.ChIP.rep1, TX1.ChIP.rep2, TX1.ChIP.rep3
Sequencing depth	TF1.ChIP.rep1: Number of reads 10,299,426; Mapped reads 4,126,095 / 40.06%;<br>TF1.ChIP.rep2: Number of reads 9,844,118; Mapped reads 3,955,724 / 40.18%;<br>TF1.ChIP.rep3: Number of reads 11,189,052; Mapped reads 4,459,606 / 39.86%;<br>TF1.Input: Number of reads 28,123,936; Mapped reads 25,003,925 / 88.91%;<br>TX1.ChIP.rep1: Number of reads 10,296,730; Mapped reads 2,269,768 / 22.04%;<br>TX1.ChIP.rep2: Number of reads 9,820,118; Mapped reads 2,172,898 / 22.13%;<br>TX1.ChIP.rep3: Number of reads 8,863,384; Mapped reads 1,972,060 / 22.25%;<br>TX1.Input: Number of reads 28,118,598; Mapped reads 25,002,697 / 88.92%;
Antibodies	Rabbit anti-FLAG primary antibody, Abcam, Cat# AB1162
Peak calling parameters	Peaks were called using MACS 1.4.2 suite with default parameters and a p-value cut off of 10-8 was used (macs14 -t ChIP.sam -c Input.sam -n name –p 10-8 -w).
Data quality	At the p-value threshold of enrichment of 10-8, all peaks enriched at FDR <5% and over 70% peaks showed > 5-fold enrichment.<br>To ensure the data quality, we overlapped the peaks called from all three-independent experiment.
Software	The ChIP-seq library was generated using the MicroPlex Library Preparation Kit v2 (Diagenode, Liège, Belgium) according to manufacturer instructions. The ChIP DNA libraries were sequenced using an Illumina HiSeq 2500 Platform by Shanghai Personal Biotechnology Cp. Ltd. Raw reads were mapped to the C. neoformans H99 genome (downloaded from http://fungidb.org/fungidb/) using the Bowtie 2 suite (version 4.1.2), as described by Langmead and Salzberg (2012). Visualization of ChIP-seq peaks was performed using IGV (Integrative Genomic Viewer, version 2.3.98).

Flow Cytometry

Plots

Confirm that:

☒ The axis labels state the marker and fluorochrome used (e.g. CD4-FITC).

☒ The axis scales are clearly visible. Include numbers along axes only for bottom left plot of group (a 'group' is an analysis of identical markers).

☒ All plots are contour plots with outliers or pseudocolor plots.

☒ A numerical value for number of cells or percentage (with statistics) is provided.

Methodology

Sample preparation	To detect reactive oxygen species (ROSs), wildtype and Cnhsf3Δ strains were separately grown overnight in fresh YPD medium until an OD600 of 0.8 was reached. DCFH-DA (Beyotime) and MitoSOX™ Red (Invitrogen) were used as ROS-staining dyes, where the former labeled intracellular ROSs, and the latter, mitochondrial ROSs. Fresh liquid YPD medium supplemented with DCFH-DA or MitoSOX™ Red according to the instructions were mixed with cells that were spun down from 1 ml of either culture. Cells were further incubated at 30°C or 40°C for 30 minutes, then pelleted using centrifugation and washed three times with PBS. To perform flow cytometry to find the number of cells with ROSs, 1 ml stained cells and 1 ml unstained cells were first diluted with PBS.
Instrument	BD LSRFortessa Cell Analyzer
Software	FlowJo version 10.0.7r2
Cell population abundance	Unstained cells are used as the control group, and the fluorescence intensity is higher than the control group as the positive cell population.
Gating strategy	Select the position with the highest cell density as the cell population for analysis of the average fluorescence intensity , and the same FSC/SSC parameter was analyzed for each group.

☒ Tick this box to confirm that a figure exemplifying the gating strategy is provided in the Supplementary Information.
